# Efficacy of resveratrol against breast cancer and hepatocellular carcinoma cell lines

**DOI:** 10.15537/smj.2023.44.3.20220768

**Published:** 2023-03

**Authors:** Nouf A. ALkharashi

**Affiliations:** *From the Department of Home Eonomy, College of Education, Prince Sattam bin Abdulaziz University, Al-kharj, Kingdom of Saudi Arabia.*

**Keywords:** MTT, resveratrol, proliferation, MCF-7, HepG2 cell line

## Abstract

**Objectives:**

To evaluate the anti-cancer effect of resveratrol on Michigan cancer foundation-7 (MCF-7) and hepatoblastoma cell line (HepG2) cells.

**Methods:**

The study was carried out at the Department of Botany and Microbiology, Prince Sattam bin Abdulaziz University, Al-kharj, Saudi Arabia, from August 2022 to October 2022. Different concentrations of resveratrol were added to the MCF-7 and HepG2 cell lines. Cell death and proliferation were measured with MTT and Trypan blue exclusion assays. Apoptosis markers were assessed by using a quantitative PCR assay (qPCR).

**Results:**

The resveratrol was shown to suppress the proliferation of MCF-7 and HepG2 cells at dose- and time-dependent. The cytotoxic effect of resveratrol was observed even at 100 μM after 24 hours. In comparison to untreated cells, resveratrol treatment reduced the viability of MCF-7 cells to roughly 57.5% with a half maximal inhibitory concentration (IC_50_) of 51.18 μM and HepG2 cells to 56.2% with an IC_50_ of 57.4 μM. Furthermore, in the tested cell lines, resveratrol was able to induce apoptosis mediated by elevated apoptosis markers.

**Conclusion:**

Resveratrol appears to be an excellent candidate agent in anticancer therapy in various human cancers.

**C**ancer continues to be the most common cause of mortality globally. In 2020, breast cancer was responsible for almost 685,000 deaths in women globally.^1^ Currently, the most widely used traditional treatment methods are surgical intervention, radiotherapy, chemotherapy, and immunotherapy, which represent conventional treatment strategies, or a combination of these options to cure cancer, shrink cancer, or stop the progression of cancer, but the availability of effective therapy for cancer is still elusive.^2,3^ Curative or primary surgery is usually carried out when cancer is found in only one part of the body. Possible complications during surgery may be caused by the surgery itself, drugs used, and the overall health status.^4^ Cancer chemotherapy agents are an important alternative to surgery and radiation to successfully treat some types of solid tumors that work primarily by interfering with either macromolecular synthesis or the function of neoplastic cells by binding with DNA and preventing RNA synthesis, leading to the death of cancer cells.^3,5^ Over the last decades, approximately 50% of all anti-cancer drugs used worldwide are either natural product derivatives or supplemented with natural products.^6^

Resveratrol is widely distributed in grapes, berries, peanuts, and red wine.^7,8^ Resveratrol appears to have the potential for the treatment of cardiovascular disease and protection against inflammatory disorders. Furthermore, emerging evidence has suggested that resveratrol has multiple anti-cancer effects, including breast, liver, prostate, colon, and lung cancer cells through several mechanisms.^7,9-13^ It has been previously shown to have growth-inhibitory activity, S-phase arrest and induction of apoptotic cell death through the intrinsic apoptotic pathway by stimulate cysteinyl aspartate proteinases (caspase) -9 and caspase-3, downregulating B-cell lymphoma 2 (Bcl-2), B-cell lymphoma-extra large (Bcl-xL), and X-linked inhibitor of apoptosis protein (XIAP) levels, and upregulating Bcl-2-associated X protein (Bax) levels in different types of cancers cells.^13-15^ It also triggers apoptotic signalling via p53-dependent or p53-independent apoptosis.^16,17^ These studies highlight that resveratrol has pro-apoptotic potential in cancer cells needs further elucidation. However, the resveratrol-induced apoptosis via a possible intracellular signalling mechanism needs further elucidation. In the current study, the anti-cancer effect of resveratrol was tested on MCF-7 and HepG2 cells.

## Methods

Ethical approval was obtained from the Standing Committee of Bioethics Research of Prince Sattam bin Abdulaziz University, Deanship of Scientific Research, Al-kharj, Saudi Arabia (approval no.: SCBR-062-2022).

Dulbecco’s Modified Eagle Medium (DMEM), and MTT solution were purchased from Gibco (Invitrogen, Grand Island, NY, USA). Resveratrol, 0.25% trypsin, 0.02% ethylenediaminetetraacetic acid (EDTA), and dimethylsulfoxide (DMSO) were purchased from Sigma-Aldrich Ltd. (Ayrshire, UK).

The cell MCF-7 and HepG2 were revived in fresh complate DMEM at 37^°^C, with 5% carbon dioxide (CO2) supply. Cells were subcultured at least once weekly.

The MCF-7 and HepG2 cells were grown at 1×103 cells/well (200 μl) in a 96-well microplate. After incubated for 24 hours at 37°C with 5% CO2, 100 μl of fresh DMEM containing different concentrations of resveratrol (100 μl, 25, 50, 100, 200, and 300 μM). Plate was then incubated for an additional 24 hours.

The MTT assay was used to estimate the cytotoxicity. Briefly, treated cells were exposed to 10 µl of MTT working solution in each well. Following 24 hours of incubation at 37°C with shaking to generate formazan crystals. The cell culture medium of cells was gently aspirated, and 100 μl/well of 100% DMSO per well was added. The absorbance was measuered after further 10 minutes- incubation at 37°C, with a microplate reader at 590 nm, ELX-808 (Biotek, USA). Cell viability (%) for each drug concentration was calculated and was determined by comparing the absorbance of the treated cells to the untreated.^18^ The half maximal inhibitory concentration (IC_50_) was defined as the concentration of the compound that causes 50% of cell proliferation inhibition and was determined by the regression probit model.

The viability of MCF-7 and HepG2 cells were carried out using Trypan blue exclusion assays, the cells were counted using an inverted light microscope.

The expression of genes coding was evaluated with resveratrol (100 μM). Ribonucleic acid isolation of cells cultured was carried out by using a RNeasy Micro Kit (QIAGEN, Hilden, Germany). The quantification of apoptosis markers in cancer cells was determined using qPCR by using one step RT² SYBR^®^ Green/ROX™ qPCR Master Mix For each sample, a master mix was prepared by mixing the following components: Go Taq Qpcr master mix (2x) (10 µl), one-step RT mix (0.4 µl), CXL (0.33 µl), F primer (0.4 µl), R primer (0.4 µl), RNase-free water (8.5 µl), and RNA template (4 µl). The mix was thoroughly mixed by gentle flicking at the bottom of the tube, centrifuged, and then transferred to the RT2 PCR Array Loading Reservoir. The one-step rRT-PCR was carried out in 7500 fast real-time PCR. The results confirmed the specificity of each primer set using melting curve analysis. The results were obtained using the 2^−ΔΔCq^ method, and delta Cq (ΔCq) values obtained for the different genes were normalized based on the value of glyceraldehyde 3-phosphate dehydrogenase (GAPDH) amplified from the same genes.^19^ The fold-change in expression was calculated as referenced to the expression of the untreated control cells. The primers used in PCR are presented in Table 1.

**Table 1:** - The primer of genes involved in apoptosis and antiapoptotic genes.^20-23^

Gene names	Primers sequence
*Caspase-3*	F: 5′-GCTGGATGCCGTCTAGAGTC-3′
	R: 5′-ATGTGTGGATGATGCTGCCA-3′
*Caspase-8*	F: 5′-AGAAGAGGGTCATCCTGGGAGA-3′
	R: 5′-TCAGGACTTCCTTCAAGGCTGC-3′
*Caspase-9*	F: 5′-ATTGCACAGCACGTTCACAC-3′
	R: 5′-TATCCCATCCCAGGAAGGCA-3′
*Bax*	F: 5′-GAGCTAGGGTCAGAGGGTCA-3′
	R: 5′-CCCCGATTCATCTACCCTGC-3′
*Bcl-2*	F: 5′-ACCTACCCAGCCTCCGTTAT-3′
	R: 5′-GAACTGGGGGAGGATTGTGG-3′
*Bcl-XL*	F: 5′-CAGAGCTTTGAACAGGTAG-3′
	R: 5′-GCTCTCGGGTGCTGTATTG-3′
*p53*	F: 5′-GCTCTGACTGTACCACCATCC-3′
	F: 5′-CTCTCGGAACATCTCGAAGCG-3′
*p21*	F: 5′-CTCAGAGGAGGCGCCATG-3′
	R: 5′-GGGCGGATTAGGGCTTCC-3′
*GAPDH*	F: 5′-CGGAGTCAACGGATTTGGTC-3′
	R: 5′-AGCCTTCTCCATGGTCGTGA-3′
Caspase: caspase protein, Bax: Bcl-2-associated X protein, Bcl-2: B-cell lymphoma 2, Bcl-XL: B-cell lymphoma-extra large, p53: Tumor protein P53, p21: Cyclin-dependent kinase inhibitor 1, GAPDH: Glyceraldehyde 3-phosphate dehydrogenase	

### Statistical analysis

The Statistical Package for the Social Sciences, version 22.0 (IBM Corp., Armonk, NY, USA) with posthoc Dennet’s test were used for data analysis. Each experiment was carried out in duplicate. Date were reported as the means ± standard deviation (SD). A *p*-value of <0.05 was considered significant.

## Results

**Figure 1:**
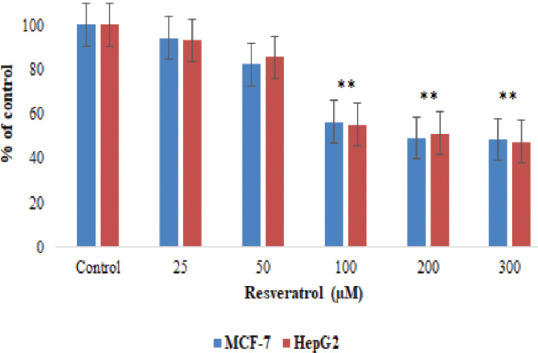
- Cytotoxicity assessment by MTT assay in MCF-7 and HepG2 cell lines treated with varius concentrations of resveratrol for 24 hours. ^**^ *p*<0.01

We found that resveratrol significantly (*p*<0.05) reduced cell growth of MCF-7 cells to roughly 57.5% with an IC_50_ of 51.18 μM and HepG2 cells to 56.2% with an IC_50_ of 57.4 μM (Figure 1).

**Figure 2:**
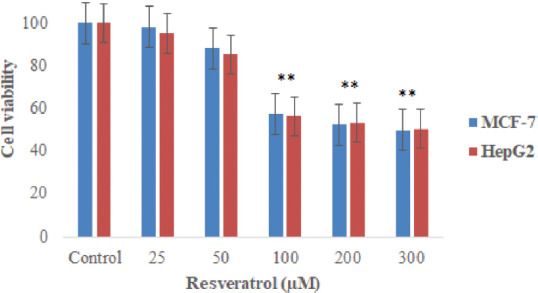
- The percentage of MCF-7 and HepG2 cells viability using a Trypan blue extraction assay for 24 hours exposure with different concentrations of resveratrol. ^**^ *p*<0.01

We also found 100 μM of resveratrol for 24 hours, decreased in percentage of viable MCF-7 cell to approximately 57.5% (*p*<0.05) and HepG2 cells to 56.2% (*p*<0.05) than untreated cells (Figure 2).

**Figure 3:**
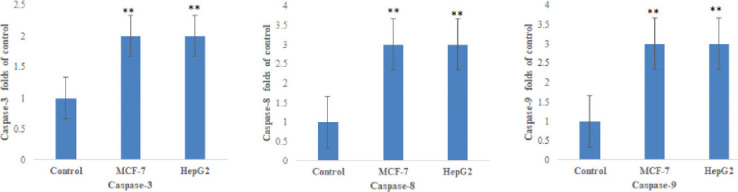
- The mRNA levels of caspase-3, caspase-8, and caspase-9 in MCF-7 and HepG2 cells. ^**^ *p*<0.01

**Figure 4:**
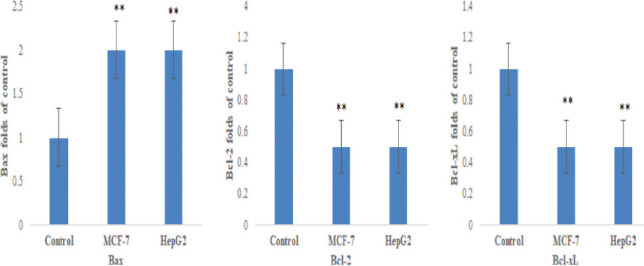
- The mRNA levels of apoptotic markers (Bax, Bcl-2, and Bcl-xL) in MCF-7 and HepG2 cells. ^**^ *p*<0.01

**Figure 5:**
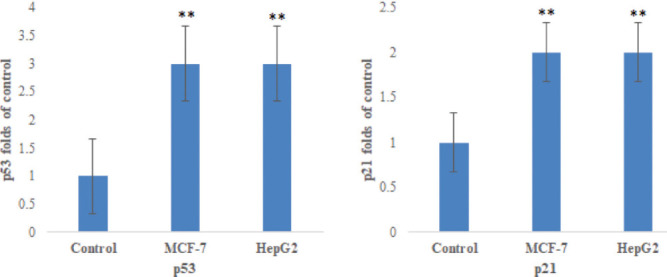
- The mRNA levels of apoptotic markers of p53 and p21 in MCF-7 and HepG2. ^**^ *p*<0.01

Figure 3 indicated that there was a significantly increase in the caspase-3, caspase-8, and caspase-9 expression in MCF-7 and HepG2 resveratrol-treated cells than untreated control cells (*p*<0.05). Resveratrol-treated MCF-7 and HepG2 cells had a highly significant Bax mRNA level and low Bcl-2 and Bcl-xL anti-apoptotic genes than control (*p*<0.05; Figure 4). Figure 5 demonstrates that resveratrol-treated MCF-7 and HepG2 cells showed higher p53 and p21 mRNA levels than control cells (*p*<0.01).

## Discussion

The limitations of conventional cancer therapies have pushed the field of chemotherapeutic drugs toward exploring other novel strategies to improve cancer therapy to inhibit cancer growth. Chemotherapeutic drugs are sometimes correlated with undesirable side effects. The plant-derived drugs were related to used as significant effective anti-cancer drugs with reduced side effects against cancer.^24^ Resveratrol, a well-known polyphenolic compound, is a nutraceutical in various plants, including grapes, peanuts, and berry fruits, and is quite famous for its association with several health benefits, such as anti-cancer potential, apoptosis, mitochondrial dysfunction, angiogenesis, antioxidant activity, and platelet aggregation inhibition.^25-27^ However, resveratrol-induced apoptosis is not fully understood.

In the cuurent study, resveratrol showed to has a significant cytotoxic effect on MCF-7 cells with an IC_50_ of 51.18 μM cells and HepG2 cells with an IC_50_ of 57.4 μM using the MTT assay. The MTT assay is a widely used method to determine the cytotoxic effects of anti-cancer compounds on cell lines.^28^ These observations are consistent with a previous study that reported that the MCF-7, HepG2, and A549 cancer cell viability was moderately inhibited by 100 μM of resveratrol for 24 hours using the MTT assay.^29^ A previous study also suggested that curcumin, resveratrol, or curcumin combined with resveratrol significantly inhibited the MCF-7 cell growth using the MTT assay after 24 hours of treatment.^30^ A 5 μg/mL resveratrol with paclitaxel (10 μg/ml) decreased the cells on HepG2 cells compared to other treatment groups.^23^ Similarly, another study reported that the MCF-7, HeLa, and HepG2 treated with resveratrol were reduced cell growth of MCF-7 cells to an IC_50_ ranging from 35.1-83.8 μM using the MTT assay.^31^ Another study reported that >1 μM resveratrol caused reduced cell proliferation in MCF-7 cells.^32^ Resveratrol was found to inhibit the HepG2 cells viablity at 24 hours with an IC_50_ value of 100 μM. These observations are in agreement with previous studies indicating that 24 hours of treatment resulted in an IC_50_ value of 60 µm resveratrol for HepG2 cells.^33^ On the other hand, resveratrol appears to have some toxic effects at high concentrations, thus, an effective strategy to decrease their chemotherapeutics concentrations may improve their anti-cancer effects.^34,35^

The apoptosis or the physiological process of cell death is characterized by distinct morphological characteristics and death.^36^ Apoptosis plays a crucial role in cancer therapy. Various studies reported targeting both the mitochondrial (intrinsic) pathway and the extrinsic pathways via death receptors on the cell surface by many anti-cancer drugs.^36-38^ The granule exocytosis pathway is an additional pathway that involves natural killer (NK) and T-cell-mediated killing of target cells.^37^ The extrinsic apoptotic pathway triggers apoptosis by the binding of ligands (namely, first apoptosis signal ligand, tumor necrosis factor, and tumour necrosis factor-related apoptosis-inducing ligand) to their death receptors.^39^ Apoptosis is also mediated by caspases. The exogenous apoptosis pathway can be activated by caspase-8 in response to the activation of cell surface death receptors.^40^ The intrinsic or mitochondrial pathway can be activated by caspase-9.^41^ Caspase-3 is involved in both exogenous and endogenous apoptosis, by interacting with caspase-8 and caspase-9, ultimately to induce cell apoptosis.^42^

In the present study, the mRNA caspase-3, caspase-8 caspase-9, p53, p21, and Bax were significantly increased in responses to 100 μM resveratrol in the MCF-7 and HepG2 cells, than control cells, whereas, there was a significantly decrease in the Bcl-2 and Bcl-xL anti-apoptotic mRNA expression than control noninfected cells. Consistent with these results reported for HepG2 cells, a previous study reported that 5 μg/mL resveratrol activated caspase-3, caspase-8, and caspase-9. Similarly, 10-80 μM of resveratrol for 48 hours inhibited the MCF-7 and MDA-MB-231 breast cancer cells by binding to and activating estrogen receptor.^43^ In addition, in vitro study used in an animal model showed that resveratrol inhibited the tumor growth at a dose of 625 mg/kg.^44^ In addition to the chemopreventive and chemoprotective effects, resveratrol possesses a very high antioxidant activity by endogenous and exogenous mechanisms.^45-48^

### Study limitations

Its flaws or shortcomings in terms of the methodology we used in our present study. Attempts to correlate protein abundance with mRNA expression level are not fully established, even though mRNA expression levels are widely employed as a proxy for estimating functional differences that occur at the protein level.^49^ Since in vitro studies are not sufficient for proving the anti-cancer effects, in vivo proof-of-concept studies can contribute to these findings.

In conclusion, resveratrol showed to have a significant cytotoxic effect on MCF-7 to roughly 57.5% with an IC_50_ of 51.18 μM and 56.2% of HepG2 cells with an IC_50_ of 57.4 μM, which may be a significant anti-cancer effect on various types of cancer such as breast cancers. Resveratrol also showed to elevate caspase-3, caspase-8, caspase-9, Bax, p53, and p21 and reduce Bcl-2 and Bcl-xL mRNA levels. Our results suggested that resveratrol could be a potentially effective approach for treating breast and liver cancer. However, synergistic anti-tumor effects of resveratrol combined with another chemotherapeutic agent are needed to reduce the IC_50_. Further, in vitro studies are not sufficient for proving the anti-cancer effects, in vivo studies can contribute to these findings.
